# Letter from the Editor in Chief

**DOI:** 10.19102/icrm.2025.16085

**Published:** 2025-08-15

**Authors:** Devi Nair


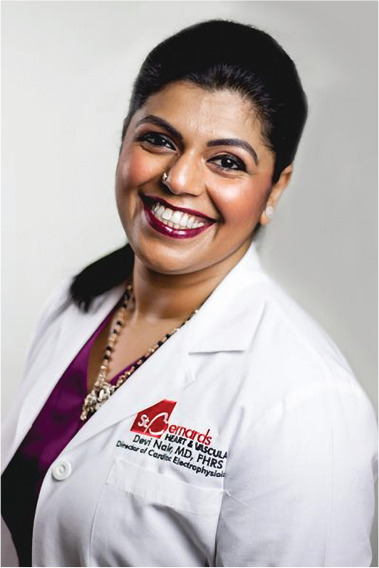


Dear Colleagues,

As we move through August, our field continues to demonstrate how science, education, and collaboration weave together to shape the future of cardiac electrophysiology. This issue of *The Journal of Innovations in Cardiac Rhythm Management* reflects both the pace of discovery and the strong spirit of community that defines our specialty.

## Advances in Science and Clinical Practice

This month’s manuscripts cover a broad and inspiring spectrum of innovation and clinical insight:

- ***Concomitant conduction system pacing during atrioventricular node ablation.*** Abuissa and colleagues^1^ present a case series of patients with long-standing atrial fibrillation undergoing simultaneous atrioventricular node ablation and His-bundle pacing via an axillary approach. Their results confirm both the feasibility and safety of this combined strategy, with durable outcomes that highlight how conduction system pacing continues to redefine physiologic pacing.
- ***Breaking the circuit in biatrial tachycardia.*** Gabarin et al.^2^ describe the management of a rare type III macro–re-entrant bi-atrial tachycardia. Using detailed bi-atrial mapping and targeted ablation, their team achieved successful termination of this challenging arrhythmia. The report underscores the importance of meticulous mapping across both atria when confronting complex circuits.
- ***The electrophysiology lab of the future.*** Cochran and Shehata^3^ offer a forward-looking perspective on how evolving technology will shape tomorrow’s electrophysiology labs. From artificial intelligence and advanced imaging to global connectivity and training accessibility, their vision lays out how integration of systems and innovation in design will enhance patient care, improve workflows, and expand global access.
- ***Catheter ablation in the elderly.*** Sawalha and colleagues^4^ provide one of the largest contemporary analyses of catheter ablation in patients over 80 years of age. Studying more than 18,000 octogenarians, they found overall complication rates to be low, though outcomes varied by arrhythmia type. This study reassures that ablation remains a viable therapy in carefully selected elderly patients, while underscoring the importance of thoughtful risk stratification.

## Nurturing the Next Generation

August was also a month rich in fellow-focused initiatives. The EP Leadership Foundations Fellows Program provided immersive education and skills training and insights into a successful transition into practice, equipping young colleagues with the tools to thrive in an evolving discipline. Similarly, the KCHRS Congress Fellows Bootcamp offered robust academic sessions, mentorship opportunities, and direct engagement with leaders in the field. These gatherings exemplify how structured educational platforms continue to elevate the training and professional growth of early-career electrophysiologists.

## HRS Advocacy and Global Integration

The Heart Rhythm Society’s strong presence at the KCHRS Congress further highlighted our community’s commitment to global collaboration. HRS engagement not only reinforced key advocacy initiatives but also underscored how deeply integrated the Society has become with the international electrophysiology community. Such partnerships remind us that, while our work is rooted in science, its true impact comes from building bridges across borders and ensuring our patients benefit from a united global voice.

## Looking Ahead

As this issue goes to press, I am struck by how innovation in technology, precision in clinical science, and investment in future generations all converge to define our field. *The Journal of Innovations in Cardiac Rhythm Management* remains proud to serve as a platform for these conversations, where data meet dialogue and where mentorship and advocacy grow alongside discovery.

We hope you find this month’s content both practical and inspiring as you continue your important work in the lab, the clinic, and beyond. Thank you for your dedication to advancing heart rhythm care, and for contributing to the momentum that makes our field one of the most collaborative and future-focused in medicine.

Warm regards,


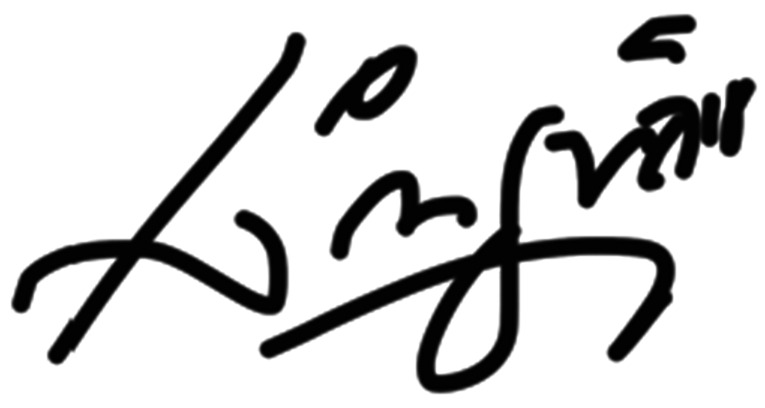


Dr. Devi Nair, md, facc, fhrs

Editor-in-Chief

*The Journal of Innovations in Cardiac Rhythm Management*

Director of the Cardiac Electrophysiology & Research,

St. Bernard’s Heart & Vascular Center, Jonesboro, AR, USA

White River Medical Center, Batesville, AR, USA

President/CEO, Arrhythmia Research Group

Clinical Adjunct Professor, University of Arkansas for Medical Sciences

Governor, Arkansas Chapter of American College of Cardiology

drdgnair@gmail.com
